# Infected Patent Urachus Presenting As Umbilical Discharge in an Adult: A Case Report

**DOI:** 10.7759/cureus.110561

**Published:** 2026-06-09

**Authors:** Andrew Roach, Joycelyn A Botelho, Ryan Muakkassa, Randall D McComb

**Affiliations:** 1 Emergency Medicine, Northeast Ohio Medical University, Akron, USA; 2 Emergency Medicine, Summa Health, Akron, USA

**Keywords:** emergency medicine, patent urachus, remnant, umbilical drainage, urology

## Abstract

The urachus is an embryologic connection between the bladder and umbilicus that typically closes by the end of the first trimester. Failure of complete obliteration results in urachal anomalies, most commonly identified during infancy and rarely persisting into adulthood. Patent urachus in adults is exceedingly uncommon and may present with nonspecific symptoms, creating diagnostic challenges in the emergency department. We report a case of a 29-year-old male who presented with acute periumbilical abdominal pain and purulent umbilical discharge. Physical examination demonstrated periumbilical tenderness, erythema, and active drainage without systemic signs of infection. Laboratory evaluation was unremarkable. Computed tomography of the abdomen and pelvis with contrast revealed a thick-walled patent urachus extending from the umbilicus to the bladder dome with surrounding inflammatory changes, consistent with superimposed infection. The patient was managed conservatively with oral amoxicillin-clavulanate, analgesia, and outpatient urology follow-up for consideration of definitive surgical excision. His symptoms improved, and he was discharged in stable condition. This case highlights the importance of maintaining a high index of suspicion for urachal pathology in young adults presenting with umbilical discharge and abdominal pain. Early recognition and appropriate imaging are essential to avoid misdiagnosis, guide management, and prevent complications such as recurrent infection or malignant transformation.

## Introduction

The urachus is a fibrous, tubular structure that connects the umbilicus to the bladder in utero [1]. It normally obliterates around week 12 of gestation, leaving behind the median umbilical ligament [1]. Failure of complete obliteration of the urachus allows urine to leak from the bladder through the umbilicus [2]. This is known as a patent urachus, which can present as persistent umbilical discharge or delayed umbilical healing. It is usually identified at birth via yellowish umbilical discharge leaking from the umbilical stump [2]. Other possible anomalies that can result from failed obliteration include urachal cysts or diverticula [1]. These anomalies are typically identified during infancy and rarely progress to adulthood.

Complications of a patent urachus include infection (abscess formation), recurrent urinary tract infections, and, rarely, malignant transformation later in life [1]. Infected urachal remnants can present with abdominal pain, fever, and umbilical erythema. Associated urogenital anomalies occur in approximately 35% of cases, including renal pelvic dilatation and vesicoureteral reflux [3]. Patients may present to the emergency department with nonspecific abdominal pain, umbilical drainage, or signs of infection, leading to misdiagnosis or delayed treatment [4]. We report a case of a patent urachus presenting in an adult with abdominal pain and purulent umbilical discharge to emphasize diagnostic considerations relevant to emergency physicians.

## Case presentation

A 29-year-old male with no significant past medical history presented to the emergency department with abdominal pain that began approximately 24 hours prior, accompanied by yellow discharge from his umbilicus that had been persisting for about a week. A Spanish-speaking telephone interpreter was used for the duration of the encounter. Further history elucidated that his symptoms began with abdominal pain the previous night that was superficial and felt like a burning pressure. He then subsequently developed recurrent drainage of pus from the site. He took Tylenol, with no alleviation of his symptoms. His symptoms persisted the following morning and continued to worsen to the point of his pain being 10/10, leading to him coming to the emergency department for evaluation. The patient denied taking any medications and denied any trauma, fevers, dysuria, nausea, vomiting, chest pain, or shortness of breath. He has no surgical history.

Vital signs were within normal limits. Physical exam revealed increased tenderness in the periumbilical region. Inspection of the umbilicus further revealed purulent drainage from the umbilicus with no obvious open wound, along with erythema in the surrounding area. The patient was given ibuprofen for pain management. Differentials at this time were broad but included intra-abdominal infection, superficial abscess, or appendicitis.

**Figure 1:**
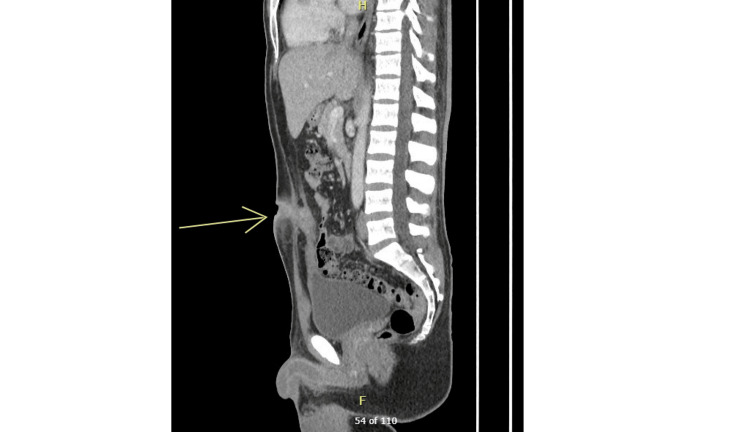
Sagittal CT image showing a patent urachus (arrow)

Laboratory evaluation yielded the following results: complete blood count (CBC), comprehensive metabolic panel (CMP), and lipase were within normal limits. Urinalysis was borderline and was significant for an increased specific gravity of 1.03 and a pH of 8.0. Urine culture yielded no growth. The patient underwent a CT scan of the abdomen and pelvis with contrast, which revealed the presence of a thick-walled patent urachus, with surrounding inflammatory fat stranding and edema, suggesting superimposed infection. The CT findings are pictured in Figure 1.

Following these radiologic findings, urology was consulted, and their recommendation was that if the patient’s pain was controlled, he could be discharged with outpatient antibiotics, specifically Augmentin, and a scheduled follow-up with them for future possible closure of the persistent urachus duct. On re-examination, the patient’s pain was improved with the ibuprofen. He was informed that he can take up to 800 mg of ibuprofen every six hours for pain control. After discussion of his condition and the need for follow-up with urology, the patient expressed understanding and was agreeable to discharge with antibiotics. The patient was discharged on a seven-day course of oral amoxicillin-clavulanate 825 mg/175 mg twice daily with urology to follow up.

## Discussion

The persistence of a patent urachus into adulthood is exceedingly rare, with the current literature indicating an incidence of approximately 1 in 5,000 adults, though the true prevalence is likely underestimated due to asymptomatic cases and delayed diagnosis [5]. In adults, infected urachal remnants frequently present with nonspecific symptoms, including abdominal pain, umbilical discharge, fever, or localized erythema. These findings may mimic more common emergency department diagnoses such as superficial skin infection, omphalitis (recent data from a nationwide Japanese study found that 16% of adults presenting with omphalitis had urachal remnants) [6], intra-abdominal infection, or abdominal wall abscess, increasing the risk of misdiagnosis or delayed recognition. In this case, the patient’s purulent umbilical drainage and abdominal pain initially raised concern for intra-abdominal pathology or superficial infection, highlighting the diagnostic challenge these anomalies can pose in the emergency setting. This case highlights the importance of maintaining a high index of suspicion for urachal pathology in young adults presenting with umbilical discharge and abdominal pain. In a retrospective study of 833,317 ED visits, symptomatic urachal remnants accounted for only 0.03% of patients presenting with abdominal pain, with 54.2% initially presenting with umbilical drainage [4]. While umbilical discharge is highly suggestive of urachal pathology, it is not pathognomonic, and imaging is essential for definitive diagnosis [4]. Our patient's presentation with purulent umbilical discharge and periumbilical pain is consistent with infected patent urachus, though initially had a broad differential and non-specific laboratory evaluation until advanced imaging was obtained.

The diagnostic workup in this case appropriately utilized CT imaging, which is the preferred modality for evaluating suspected urachal pathology in adults. CT demonstrates a patent urachus as an elongated tubular structure extending from the umbilicus to the anterosuperior bladder dome, with infected remnants showing thick walls, surrounding inflammatory fat stranding, and edema, as was seen in this case [7,8]. The characteristic midline location and connection to the bladder dome help distinguish urachal pathology from other causes of periumbilical inflammation. Ultrasound can also be diagnostic, particularly for urachal cysts, though CT provides superior anatomic detail and evaluation of complications [1,9]. The management of symptomatic urachal remnants in adults has evolved considerably, with ongoing debate regarding optimal timing and approach. Symptomatic urachal remnants generally require surgical excision to prevent recurrent infection and eliminate the theoretical risk of malignant transformation [10]. Pathological studies have shown that 65% of excised urachal remnants contain epithelial lining, which over time could undergo malignant transformation [10]. The acute management of infected urachal remnants varies based on the severity of infection and patient stability. In this case, the patient was managed conservatively with outpatient antibiotics and scheduled urology follow-up, which represents an appropriate level of treatment for clinically stable patients with controlled pain and no signs of systemic infection or abscess requiring drainage. The choice of amoxicillin-clavulanate provides coverage for common skin flora, enteric organisms, and urinary pathogens, which are frequently implicated in infected urachal anomalies.

This case underscores several important lessons for emergency physicians. First, urachal pathology should be included in the differential diagnosis of young adults presenting with umbilical discharge, periumbilical pain, or recurrent urinary tract infections. Second, CT imaging is essential for diagnosis and should be obtained when urachal pathology is suspected, as physical examination and laboratory studies are often nonspecific [4]. Recognition of urachal anomalies as a potential underlying etiology can prompt timely imaging, appropriate antibiotic therapy, and referral for definitive surgical management, thereby reducing the risk of complications associated with delayed diagnosis. The management approach in the ED should focus on controlling acute infection, providing adequate analgesia, and ensuring appropriate urologic follow-up. Patients with well-controlled symptoms and no evidence of abscess or systemic infection can be safely discharged with oral antibiotics and close follow-up, as demonstrated in this case. However, patients with signs of sepsis, large abscesses, or inability to tolerate oral intake may require admission for intravenous antibiotics and possible percutaneous or surgical drainage.

## Conclusions

This case illustrates one possible presentation of an infected patent urachus in an adult and highlights the diagnostic and management challenges faced by emergency physicians. While rare, urachal remnants should be considered in the differential diagnosis when symptoms include umbilical discharge and periumbilical pain. CT imaging is essential for diagnosis, and symptomatic patients generally require definitive surgical excision following resolution of acute infection. Urologic follow-up is likewise a key part of treatment to ensure definitive management and monitoring for potential complications.
